# Retinoic Acid Signaling: A New Piece in the Spoken Language Puzzle

**DOI:** 10.3389/fpsyg.2015.01816

**Published:** 2015-11-26

**Authors:** Jon-Ruben van Rhijn, Sonja C. Vernes

**Affiliations:** ^1^Department of Language and Genetics, Max Planck Institute for PsycholinguisticsNijmegen, Netherlands; ^2^Molecular Neurophysiology Group, Department of Cognitive Neuroscience, Radboud University Medical CenterNijmegen, Netherlands; ^3^Donders Institute for Brain, Cognition and Behaviour, Radboud UniversityNijmegen, Netherlands

**Keywords:** retinoic acid, FoxP2, synaptic plasticity, development, motor skills, striatum, dopamine receptor

## Abstract

Speech requires precise motor control and rapid sequencing of highly complex vocal musculature. Despite its complexity, most people produce spoken language effortlessly. This is due to activity in distributed neuronal circuitry including cortico-striato-thalamic loops that control speech–motor output. Understanding the neuro-genetic mechanisms involved in the correct development and function of these pathways will shed light on how humans can effortlessly and innately use spoken language and help to elucidate what goes wrong in speech-language disorders. *FOXP2* was the first single gene identified to cause speech and language disorder. Individuals with *FOXP2* mutations display a severe speech deficit that includes receptive and expressive language impairments. The neuro-molecular mechanisms controlled by *FOXP2* will give insight into our capacity for speech–motor control, but are only beginning to be unraveled. Recently FOXP2 was found to regulate genes involved in retinoic acid (RA) signaling and to modify the cellular response to RA, a key regulator of brain development. Here we explore evidence that FOXP2 and RA function in overlapping pathways. We summate evidence at molecular, cellular, and behavioral levels that suggest an interplay between FOXP2 and RA that may be important for fine motor control and speech–motor output. We propose RA signaling is an exciting new angle from which to investigate how neuro-genetic mechanisms can contribute to the (spoken) language ready brain.

## Speech And Spoken Language

**FIGURE 1:**
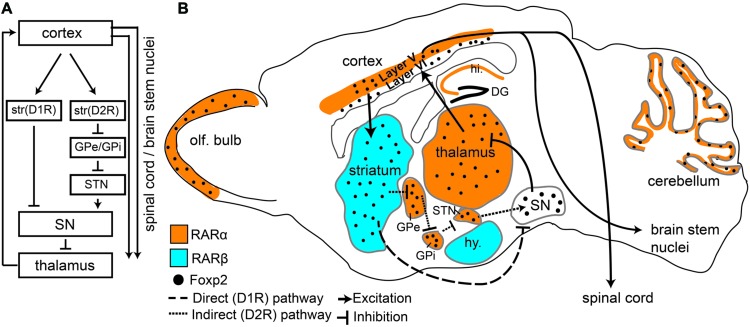
**Foxp2 and retinoic acid receptors (RARs) show overlapping expression patterns in motor associated circuitry.** **(A)** An overview of the direct and indirect pathways represented in the sagittal view showing connectivity between different regions. Dopamine receptor type 1 (D1R) and Dopamine receptor type 2 (D2R) expressing cells in the striatum are separated to highlight direct and indirect pathways. **(B)** Sagittal Schematic of the mouse brain showing that Foxp2, RARα, and RARβ are all expressed in motor associated circuitry. RARα and RARβ are expressed in distinct regions, but each receptor partially overlaps with Foxp2. RARα and Foxp2 can be found in deep layers of the cortex, thalamus, subthalamic nucleus (STN), the internal (GPi) and external (GPe) globus pallidus, cerebellum, and olfactory bulbs (OB). Foxp2 and RARβ overlap in the striatum. RARα shows non-overlapping expression in the hippocampus (hi.), RARβ in the hypothalamus (hy), and Foxp2 in the substantia nigra (SN). Connectivity between regions involved in motor processing (including outputs to brain stem nuclei and spinal cord) is shown by solid lines. The direct (excitatory) and indirect (inhibitory) pathways, which are the two outputs from the striatum, are shown by dashed lines.

Speech is the primary modality by which humans use language, and human orofacial morphology is uniquely suited to the production of intricate vocalizations needed for spoken language (Lieberman, 2007). The orofacial musculature is one of the most complex muscle systems in the body and in order to successfully produce meaningful speech these muscles must be controlled and coordinated in rapid sequences involving distributed neuronal circuitry. This motor activity is generated in several neural loops that select appropriate actions and generate the necessary motor patterns. One crucial circuit, the cortico-basal ganglia loop, sends activity from the motor cortex to the striatum (a component of the basal ganglia) where activity is integrated. Subsequently, outputs from here modulate activity in several thalamic nuclei. Activity from the thalamus is then sent back to the motor cortex, where a specialized population of output neurons organizes the complex thalamocortical inputs (Kravitz and Kreitzer, 2012; Calabresi et al., 2014). These cortical output neurons send the information, via the pyramidal tract, to motor neurons directly controlling muscle tissue. These neurons are either located in the spinal cord (controlling limb and body movements), or in the brainstem’s cranial nerve nuclei (controlling facial and vocal tract movements). An illustration of the cortico-basal ganglia loop (in the rodent brain) is given in **Figure 1A**. Proper connectivity within this pathway is necessary to enable the precise outputs needed for orofacial muscle control.

The striatum can be seen as a central hub within the motor pathway, making it one of the most intriguing regions in which to investigate properties of motor circuitry and orofacial control. Striatal activity is especially important for fine motor behavior and motor skill learning (Doyon et al., 2003) and cortical and subcortical circuitry, including the striatum, has been established as highly important for speech–motor control (Lieberman, 2002). Furthermore, increased activation of the basal ganglia (which incorporates the striatum) has been shown via functional brain imaging (fMRI) in specific speech–motor language tasks (Wildgruber et al., 2001; Booth et al., 2007). Lastly, morphological changes in the striatum have been described in individuals with speech problems such as stuttering (Craig-McQuaide et al., 2014) and non-fluent aphasia (Ogar et al., 2007).

The principal cell type in the striatum is the medium spiny neuron (MSN), which makes up approximately 98% of all striatal cells (Kemp and Powell, 1971; Huang et al., 1992; for review, see Kreitzer and Malenka, 2008). MSNs can be further divided into two categories of neurons that have different connectivity and opposing functions: dopamine receptor type 1 (D1R) and dopamine receptor type 2 (D2R) expressing cells (**Figure 1A**). D1R expressing MSNs connect to thalamic nuclei via the “direct pathway” which results in excitation of the motor cortex. D2R expressing MSNs form an “indirect pathway” that connects to the thalamus via multiple subcortical regions leading to inhibition of the thalamus and thus reduced cortical input (**Figure 1A**), (Albin et al., 1989; Kravitz and Kreitzer, 2012; Calabresi et al., 2014). This balance between excitation (resulting in more movement) and inhibition (less movement) is crucial for coordinated motor function (Calabresi et al., 2014) including fine orofacial motor control.

In order to unravel the fundamental components that enable humans to effortlessly use spoken language, we will need to understand the neuro-genetic mechanisms involved in establishment, function, and maintenance of speech–motor pathways.

## Spoken Language And Foxp2

A breakthrough in speech and language genetics came with the identification of the first gene to cause a speech/language disorder: *FOXP2* (Lai et al., 2001). Mutations in *FOXP2* were found in a large pedigree known as the KE family (Hurst et al., 1990; Fisher et al., 1998; Lai et al., 2001). Affected family members were diagnosed with a severe speech impairment known as developmental verbal dyspraxia (also known as childhood apraxia of speech; OMIM: 602081) and carried a mutation in one copy of their *FOXP2* gene. In addition to speech impairments, affected family members demonstrated receptive and expressive language problems (Watkins et al., 2002a). Although rare, *FOXP2* mutations have been found in a number of unrelated families and individuals with similar speech/language phenotypes (MacDermot et al., 2005; Feuk et al., 2006; Shriberg et al., 2006; Lennon et al., 2007; Palka et al., 2012; Rice et al., 2012; Zilina et al., 2012; for review, see Bacon and Rappold, 2012). In depth investigations of the KE family phenotype indicated a severe impairment in orofacial praxis tasks (Vargha-Khadem et al., 1995; Lai et al., 2001; Watkins et al., 2002a). In addition, impairments in language production tasks (e.g., phoneme addition, word repetition) were found between control and affected individuals (Vargha-Khadem et al., 1995). Different aspects of speech are thus impaired in KE family members (Watkins et al., 2002a). Orofacial praxis deficits underlie impaired lexicon building and subvocal (internal) speech representations which can affect irregular verb grammar (Doyon et al., 2003) and rule based grammar learning (Ullman, 2001). Thus, some of the language impairments in the KE family could be related to the core speech production deficits observed.

FOXP2, and its murine homolog Foxp2, are found across many regions of the developing and postnatal brain (FoxP2 will be used when referring to both species). Intriguing is the high expression of FoxP2 throughout the mouse and human cortico-striato-thalamic motor circuitry (Lai et al., 2003). During early development FoxP2 is broadly expressed in these regions, but in later developmental and postnatal stages expression becomes more restricted (**Figure 1B** depicts Foxp2 expression in the postnatal mouse brain). In adults, Foxp2 is limited to deep layer cortical neurons (layer 5 motor cortex and layer 6 throughout; Ferland et al., 2003; Morikawa et al., 2009; Hisaoka et al., 2010; Tomassy et al., 2010; Reimers-Kipping et al., 2011; Tsui et al., 2013). Within the striatum, Foxp2 is highly expressed in both types of MSN, though more commonly in D1R MSNs compared to D2R neurons (Vernes et al., 2011). Corresponding with its expression pattern, imaging studies have shown humans with *FOXP2* mutations display structural and functional differences in motor areas. Affected members of the KE family showed structural gray matter volume differences in the motor cortex and striatum (Watkins et al., 2002b). Furthermore, functional imaging studies showed an underactivation of the striatum and altered cortical activation (including speech/motor areas such as the left anterior insular cortex) during word generation and word repetition tasks (Liegeois et al., 2003).

Converging evidence from FoxP2 expression pattern studies and phenotypic characterization of human mutations suggests that FOXP2 may play an important role in the development of the speech–motor pathway. The high expression of Foxp2 in a specific subset of neurons (D1R MSNs) in the striatum indicates a functional specificity related to motor tasks requiring the striato-thalamic connections of the direct pathway. Malfunctions within this pathway could ultimately affect aspects of the motor circuitry related to fine motor control and contribute to the observed speech–motor deficit in humans.

## Foxp2 As A Molecular Entry Point Into Speech–Motor Pathways

FoxP2 is a transcription factor; its molecular function is to regulate the expression of other genes, switching them on or off in a temporally and spatially controlled manner. FoxP2 has been shown to regulate 100s of different genes involved in processes crucial to brain development and function, ranging from neurogenesis and migration, to neurite outgrowth and synaptic activity (Spiteri et al., 2007; Vernes et al., 2007, 2011; Konopka et al., 2009; Devanna et al., 2014). Recently, evidence has suggested that FOXP2 regulates a number of genes involved in the retinoic acid (RA) signaling pathway (Devanna et al., 2014). RA is a vitamin-A derivative essential to mammalian development. Disruption of the RA signaling pathway (caused by genetic disruptions or dietary deficiencies) can have severe consequences during development and adulthood (Holson et al., 1997; Krezel et al., 1998)

Retinoic acid induces genetic and morphological changes in cells. When neuronal precursors (cells that generate neurons during development) differentiate into neurons they switch on genes normally found in mature neurons, stop dividing and grow long processes known as neurites (Siegenthaler et al., 2009; Korecka et al., 2013). We previously compared how neuron-like cells with or without FOXP2 responded to RA and found that cells showed stronger genetic and morphological changes in response to RA if FOXP2 was present (Devanna et al., 2014). In addition we discovered that FOXP2 changed the expression of RA receptors – proteins that directly control the cellular response to RA (Devanna et al., 2014). Of particular interest, FOXP2 upregulated retinoic acid receptor β (RARβ) and a number of other genes involved in transport or modification of RA were also transcriptionally regulated (e.g., RORβ, CRABPII, and ASCL1). These experiments suggest an intriguing link between FOXP2 and the RA pathway, in which FOXP2 seems to contribute to or modify the cellular response to RA.

Given the importance of the RA pathway for development, this raises new questions about how FOXP2 might mediate its effects on brain and neural circuit development. Could the relationship between FOXP2 and the RA pathway be relevant for (1) normal motor circuitry development and function, and/or (2) effects of FOXP2 dysfunction in patients? To address these questions, we need to understand how FoxP2 and the RA pathway might interact, and in what way FoxP2 mutations might affect the RA pathway on a cellular, functional and behavioral level.

## Ra, Foxp2, And Motor Behavior

Retinoic acid is a key compound during embryogenesis, affecting a multitude of critical developmental pathways. Precise control of RA levels is essential for normal brain development as either an excess or a deficiency of RA results in widespread adverse effects on the brain.

Gestational treatment of rats with excess RA results in behavioral deficits in learning, memory and motor function (Holson et al., 1997). Rats treated with excess RA displayed poor generalized motor control including impairments in the ‘righting reflex’ (the ability to return to upright position), and the ability to sit only on the back paws. In addition, gestationally treated adult rats showed problems with learning and memory, such as decreased learning rates in a water filled T maze (Butcher et al., 1972; Holson et al., 1997). Rats lacking dietary vitamin A (of which RA is a metabolite) also perform poorly on motor learning and motor performance tasks (Carta et al., 2006). Furthermore, mice engineered to lack a key facilitator of RA signaling (RARβ) develop severe locomotion deficits and are highly impaired on motor learning tasks (Krezel et al., 1998).

The displayed motor deficits are similar to phenotypes observed in mouse models of Foxp2 dysfunction. Mouse models of two well characterized patient mutations of FOXP2 have been created that have comparable phenotypes. One mouse model reflects the R553H missense mutation found in the KE family (Lai et al., 2001). The second mouse model mirrors an early stop codon in exon 7 introduced by a non-sense mutation that leads to a loss of FOXP2 protein in an independent family with speech/language disorder (MacDermot et al., 2005; Groszer et al., 2008). Mice that have a homozygous Foxp2 mutation show severe general motor impairments, reminiscent of animals treated with excess RA. However these Foxp2 homozygous mutants do not survive beyond 3–4 weeks after birth, possibly due to a requirement for Foxp2 in other organs such as the lungs or heart (Groszer et al., 2008). In mice where a single copy of Foxp2 is affected (as per the heterozygous state of the mutations observed in patients) general motor control is normal but motor learning is impaired (Groszer et al., 2008; French et al., 2012). This more subtle phenotype closely resembles the motor learning phenotype observed in RA deprived rats (Carta et al., 2006). For an overview of the different phenotypes exhibited by Foxp2 mutation, RAR mutation, and RA treatment, see **Table 1**.

**Table 1:** Overview of phenotypes described in Foxp2 mutation, retinoic acid receptor (RAR) mutation and RA excess/depletion treatments.

Deficit		Foxp2 mutation		RA receptor mutation	RA excess/depletion
				
		Homozygous	Heterozygous		
**Development**
	Embryogenesis defects	**–**	**–**	**–**	+
	Lethality	++	–	+	++
	Aberrant basal ganglia development	++	+	++	++

**Cellular**
	Basal ganglia cell identity defects	NT	–	+	++
	Decreased neurite growth and branching	++	NT	NT	NT
	Aberrant neuronal activity in striatum	++	++	–	+
	Unable to induce LTD	NT	++	++	NT
	Unable to induce LTP	NT	NT	++	NT

**Behavior**
	General motor control deficits	++	–	++	++
	Motor learning deficits	N/A	+	++	++
		(postnatal lethality)			(postnatal treatment)
	Spatial learning deficits	N/A	NT	+	+
		(postnatal lethality)			


*–, no effect; +, mild effect; ++, strong effect; N/A, not applicable; NT, not tested.*

## Foxp2 And Ra Signaling Affect Neuronal Function

In addition to the behavioral deficits, vitamin A depri vation/supplementation adversely affects striatal development and function. Cells in the developing lateral ganglionic eminence (the precursor region of the striatum) do not differentiate into the appropriate neuronal subtypes when RA signaling is blocked (Toresson et al., 1999; Chatzi et al., 2011). However restoring RA levels rescued this phenotype and resulted in normal differentiation into appropriate neuronal cell types (Chatzi et al., 2011). Separately, mice engineered to knockout the *RAR*β gene display gross morphological striatal defects including impaired neurogenesis and deficits in acquiring proper neuronal identities (Liao et al., 2008). Lastly, chronic postnatal vitamin A supplementation has been linked to oxidative cell toxicity in the striatum (de Oliveira et al., 2007).

Foxp2 also contributes to striatal cell morphology and function. Foxp2 mutant neurons exhibit reduced neurite growth and branching in primary striatal cultures (Vernes et al., 2011) and the *in vivo* striatum displays aberrant neuronal activity. Mice with a heterozygous Foxp2 mutation showed unusually high activity in the dorsomedial striatum during active motor behavior (French et al., 2012). This suggests striatal cells can no longer properly modulate their activity following input from motor areas when lacking Foxp2. Moreover, the increased striatal activity normally seen when animals perform motor learning tasks was absent in mutant mice. Instead, a decrease in firing rate was seen, again suggesting aberrant modulation of responses to cortical and/or thalamic input (French et al., 2012). Additionally, extracellular measurements on striatal brain slices from heterozygous Foxp2 mutant animals show these cells fail to respond to induction of long term depression (LTD; Groszer et al., 2008). An inability to induce long term plasticity [either LTD or long term potentiation (LTP)] has debilitating consequences as scaled activity (plasticity) is necessary for circuits to properly regulate their input and output. Synaptic long term plasticity changes underlie information storage and are necessary for learning and memory (Novkovic et al., 2015; Zhu et al., 2015). Interestingly, in the striatum, synaptic plasticity has been strongly linked to motor learning (Dang et al., 2006; Kreitzer and Malenka, 2007). Defects specifically related to striatal LTD and LTP are known to affect procedural motor learning and the acquisition of new motor paradigms (Gubellini et al., 2004).

Aberrant induction of synaptic scaling has also been found in mice following acute RA depletion, which results in a complete lack of hippocampal LTP or LTD (Misner et al., 2001). This phenotype was specific to RA depletion and was reversible, as vitamin A supplementation rapidly restored normal synaptic plasticity (Misner et al., 2001). At a molecular level, RA signaling is mediated by the action of RA receptors (RARs; RARα, RARβ, and RARγ) and similar plasticity defects have been shown for mice lacking RARα (Sarti et al., 2012) or RARβ (Chiang et al., 1998). Hippocampal cells from these mice fail to establish LTD when subjected to low frequency stimulation – the paradigm necessary to induce LTD in the hippocampus. By contrast, excess RA induced the reverse effect in cultured hippocampal slices, where increased excitatory activity was observed (Aoto et al., 2008). It is not yet known if RA signaling affects synaptic plasticity in the striatum. However, the similarity in synaptic activity phenotypes between Foxp2-, RARα-, and RARβ-deficient animals (albeit focusing on different brain regions) does indicate these transcription factors may play a role in similar intracellular pathways regulating neuronal activity and synaptic plasticity.

The aforementioned plasticity (LTD and/or LTP) deficits in Foxp2, RARα, and RARβ mutant animals suggests an improper reaction of neuronal circuits to changes in external input. Induction of LTD or LTP leads to a decrease or an increase, respectively, in the amount of glutamate receptors (of the AMPA-receptor class) at the synaptic membrane (Seidenman et al., 2003; Briand et al., 2014; for review, see Luscher and Huber, 2010). This change in AMPA receptor abundance modifies the response strength of a cell when it is excited. The change in stimulus–response strength is transient, and in time the normal AMPA receptor distribution will be restored, returning synaptic responses to normal levels. RA treatment of hippocampal cultures has shown an increase of AMPA receptors on the cell surface (Aoto et al., 2008), but no data on the striatum is currently present. The shared synaptic plasticity defect following disruption of RA signaling pathways or Foxp2 mutation does suggest that they both may influence receptor abundance or localization at the synapse in the striatum, an intriguing area for further study.

A thorough investigation of the mechanisms leading to LTD and LTP deficits resulting from RA/RAR and Foxp2 malfunction will be necessary to understand if they function in the same pathways. Understanding the molecular mechanisms underlying striatal function, especially related to complex motor circuitry function, will lead to a better understanding of striatal speech–motor control.

## Molecular Links Between Rars And Foxp2

Retinoic acid receptors canonically function as transcription factors, regulating genes responsible for directing normal embryogenesis and brain development. Interestingly, FoxP2 and RARs share some of the same target genes (Balmer and Blomhoff, 2002; Delacroix et al., 2010; Devanna et al., 2014). RARs are highly expressed in the brain (Krezel et al., 1999) and are present throughout embryonal development (Mollard et al., 2000), postnatal development (Wei et al., 2011), and in adults (Krezel et al., 1999; Zetterstrom et al., 1999). Notably high expression of RARs can be found throughout the motor circuitry, including cortical, striatal, and multiple thalamic regions (Krezel et al., 1999), (**Figure 1B**). We focus on two key receptors found in the motor circuitry: RARα and RARβ. RARα is found in layer 5 of the cortex and in the thalamus – both regions that overlap with murine Foxp2 expression (Krezel et al., 1999; Zetterstrom et al., 1999; Ferland et al., 2003; Lai et al., 2003; Hisaoka et al., 2010). Interestingly, Foxp2 only overlaps with RARα in the motor cortex layer 5, because Foxp2 expression is largely restricted to layer 6 of other mature cortical areas. RARβ is strongly expressed only in the striatum, another site where Foxp2 expression is highest (**Figure 1B**). Notably, FOXP2 has been shown to directly drive RARβ expression in human cells (Vernes et al., 2007; Devanna et al., 2014), although this is yet to be shown in the striatum. This high level of overlap, combined with shared target genes and molecular interactions, strongly supports interplay between FoxP2 and RARs in motor pathways.

## Concluding Remarks

In addition to its canonical role during embryogenesis, studies described here suggest RA signaling plays a specific role in the development and function of striatal motor circuitry and may link to FoxP2 function. Disruption of the RA pathway results in strikingly similar phenotypes to FoxP2 mutation on multiple levels, which suggests a potential mechanistic interaction. FoxP2 and RARs can regulate some common target genes, affect similar cellular phenotypes and show highly overlapping expression patterns in the cortico-striato-thalamic motor circuitry. In the striatum, aberrant function of Foxp2 and RA signaling contributes to altered development and, in the case of mutations of mouse Foxp2, altered synaptic plasticity similar to that seen in the hippocampus of RARα mutant animals. Given that RARβ is predominantly expressed in the postnatal striatum, it seems likely that its disruption will also affect striatal plasticity, however, this is yet to be experimentally determined. Lastly, animals with mutated Foxp2 or RA signaling defects show comparable motor control/learning impairments. Thus at multiple levels (molecular, cellular, circuit, and behavioral) there is evidence that interplay between FoxP2 and RA signaling may facilitate proper development and function of motor circuitry. This evidence from mice is strengthened by findings in songbirds which show both FoxP2 and RA influence song learning by acting in circuits that have parallels with human vocal-motor pathways (Haesler et al., 2007; Wood et al., 2008). In the future it will be of great value to understand if these signaling cascades interact to influence neuronal mechanisms related to song learning or speech–motor control, and if RA signaling deficits are involved in aberrant speech–motor development in humans. The capacity for human speech and spoken language is dependent on multiple molecular and neural building blocks. With the link between FoxP2 and RA signaling, a new block has been suggested, giving us new opportunities to investigate the evolution and development of the (spoken) language ready brain.

## Conflict of Interest Statement

The authors declare that the research was conducted in the absence of any commercial or financial relationships that could be construed as a potential conflict of interest.
